# MicroRNA and Messenger RNA Expression Profiles in Canine Mammary Gland Tumor

**DOI:** 10.3390/ijms24032618

**Published:** 2023-01-30

**Authors:** Eun Pyo Kim, Giup Jang, Jin-Wook Kim, Sang Wha Kim, Heaji Chung, Yun Jung Yang, Wan Hee Kim, Geon A Kim

**Affiliations:** 1 Department of Theriogenology and Biotechnology, Research Institute for Veterinary Science, College of Veterinary Medicine, Seoul National University, Seoul 08826, Republic of Korea; 2 Research and Development Center, Einocle Inc., Seoul 02841, Republic of Korea; 3 Research and Development Center, ROKIT Healthcare Inc., Seoul 08512, Republic of Korea; 4 Department of Microbiology and Immunology, College of Medicine, Seoul National University, Seoul 03080, Republic of Korea; 5 Institute of Endemic Disease, College of Medicine, Seoul National University, Seoul 03080, Republic of Korea; 6 Department of General Surgery, College of Veterinary Medicine, Seoul National University, Seoul 08826, Republic of Korea; 7 Department of Biomedical Laboratory Science, School of Healthcare Science, Eulji University, Uijeongbu 11759, Republic of Korea; 8 Department of Senior Healthcare, Eulji University, Uijeongbu 11759, Republic of Korea

**Keywords:** microRNA, canine, mammary gland, tumor, cancer, expression

## Abstract

Canine mammary gland tumor (CMT) is the most frequently diagnosed neoplasm in intact female dogs. As prognosis depends on the malignancy of tumors and metastasis levels, early and accurate diagnosis are crucial for prolongation of life expectancy. The genetic similarity of dogs with humans in addition to environmental and physiological similarities make them ideal models for the study of cancer. In this study, we analyzed differentially expressed microRNAs followed by RNA-Seq to investigate the alterations in mRNA levels based on the malignancy (benign, malignant) and the biopsy locations (tumors, surrounding normal tissues). We identified multiple breast cancer-related genes regardless of malignancy. We found cfa-miR-503 to be the only miRNA that showed altered expression in response to malignancy in CMTs. Although further validation is needed, cfa-miR-503 could be used as a potential diagnostic biomarker as well as a potential RNA-based anti-tumor drug in malignant CMTs.

## 1. Introduction

Cancer is the most common cause of death in dogs [1]. Among various cancers, canine mammary gland tumor (CMT) is the most frequently occurring tumor in intact female dogs [2,3,4]. Approximately 50% of the CMT cases lead to malignancy [5]; owing to its high incidence and mortality rates, CMT is of significance in canine medicine. To date, several studies have focused on CMT owing to its highly complex pathogenicity [2,6,7,8,9].

In addition to its clinical significance to dogs, studies on canine cancer are also applicable to medical research targeting human diseases, as dogs have been considered to be ideal animal models [1]. Dogs contract clinically similar diseases to humans; they also share similar organ sizes with humans [10,11]. The fact that dogs share similar living spaces with their human owners, minimizes gaps in environmental factors between the two [10,11]. Since dogs age faster than humans and usually experience minimal genetic variations owing to breeding, they are valuable animal models of human disease [12]. The variety of studies conducted on dogs to date have continuously proven the value of dogs as model species connecting mice and humans.

Dogs have greater genetic similarity with humans than mice [11]. About half of the hereditary diseases in dogs are remarkably similar to those in humans [11]. CMT in dogs share similarities with breast cancer (BC) in humans: In dogs, diverse genes including Tumor protein 53 (p53), Breast Cancer1 (BRCA1), Breast Cancer 2 (BRCA2), Phosphatase and Tensin Homolog (PTEN), as well as E-cadherin and Serine/Threonine kinase11 (STK11) are known contributors of CMT [13], which have also been attributed to human BC.

The microRNAs (miRNAs), also known as small non-coding RNA, are usually composed of about 22 nucleotides. In addition, miRNAs play key roles in post-transcriptional gene silencing by pairing with complementary messenger RNA in diverse animal clades [14]. Thanks to their functional characteristics, miRNA studies have been actively conducted to gain new insights in human diseases. In BC, studies involving miRNA include using it as a biomarker, profiling miRNA for cancer diagnosis, as well as developing miRNAs as tools to understand disease prognosis, therapy response, and resistance mechanisms [15,16,17].

Mature canine miRNAs show high homology to those of humans, which opens possibilities for comparative studies [18]. The importance of miRNA in canine cancer studies has been identified to be of significance leading to a number of studies [19,20,21,22]. A miRNA study on CMT, targeting 10 types of human oncomirs identified nine miRNAs (let-7f, miR-15a, miR-16, miR-17-5p, miR-21, miR-29b, miR-125b, miR-155, miR-181b) which showed expression patterns similar to miRNAs in human BC [23]. However, systemic miRNA profiling of CMT is yet to be conducted.

The objective of this study was to identify the expression patterns of miRNAs in CMT tissues. Benign and malignant CMTs determined based on histopathology and metastasis patterns would be compared with miRNA expression profiles of normal tissues. Similar comparative analysis would also be performed between benign and malignant CMTs. Differentially expressed miRNAs (DE-miRNAs) from the above comparative analyses would be listed, and their functions and expression patterns in both dogs and humans would be discussed based on the existing literature.

## 2. Results

**Figure 1:**
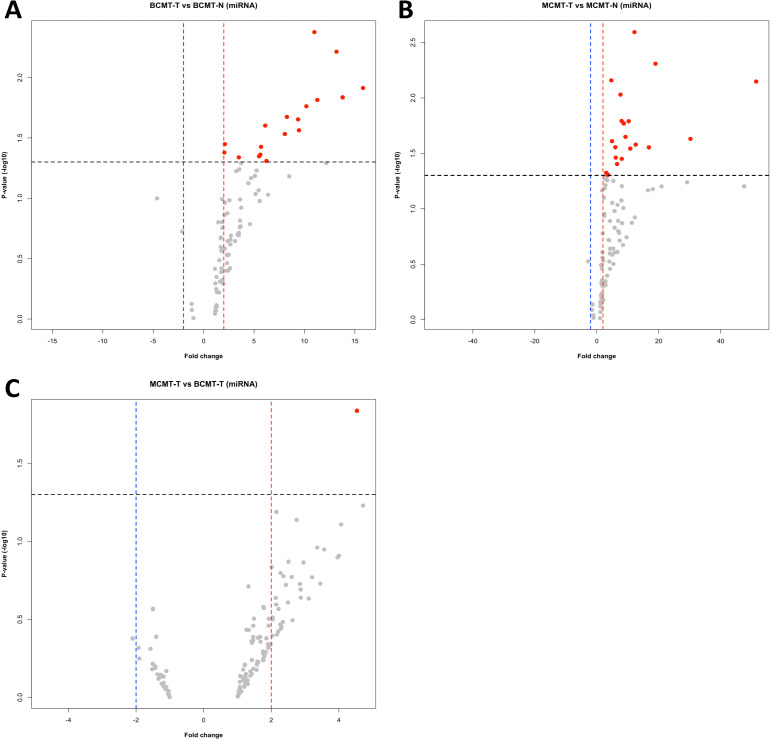
Volcano plot in canine mammary gland tumor for array-based miRNA. Red dots indicate upregulated miRNAs and grey dots indicate unsignificant miRNAs. Red and blue dotted line are threshold of fold change and black dotted line is threshold of *p*-value. (**A**) BCMT-T vs. BCMT-N; (**B**) MCMT-T vs. MCMT-N; (**C**) MCMT-T vs. BCMT-T (Figure 2).

DE-miRNAs based on microarray were identified using three comparisons: (1) BCMT-T vs. BCMT-N, (2) MCMT-T vs. MCMT-N, and (3) MCMT-T vs. BCMT-T; T means tumor and N means normal. The cut-off values are |fold change| ≥ 2, *p*-value < 0.05 (*t*-test), and DABG *p*-value < 0.05. DABG *p*-value was computed based on the probability that the signal intensity is part of the null distribution. It could be useful in the detection and removal of low intensity signals. Eighteen upregulated miRNAs in BCMT-T vs. BCMT-N, twenty upregulated miRNAs in MCMT-T vs. MCMT-N, and one upregulated miRNA in MCMT-T vs. BCMT-T were identified. Downregulated miRNAs were not detected in all comparisons. Figure 1 shows the volcano plot for identifying DE-miRNAs of each comparison based on fold change and *p*-value.

**Figure 2:**
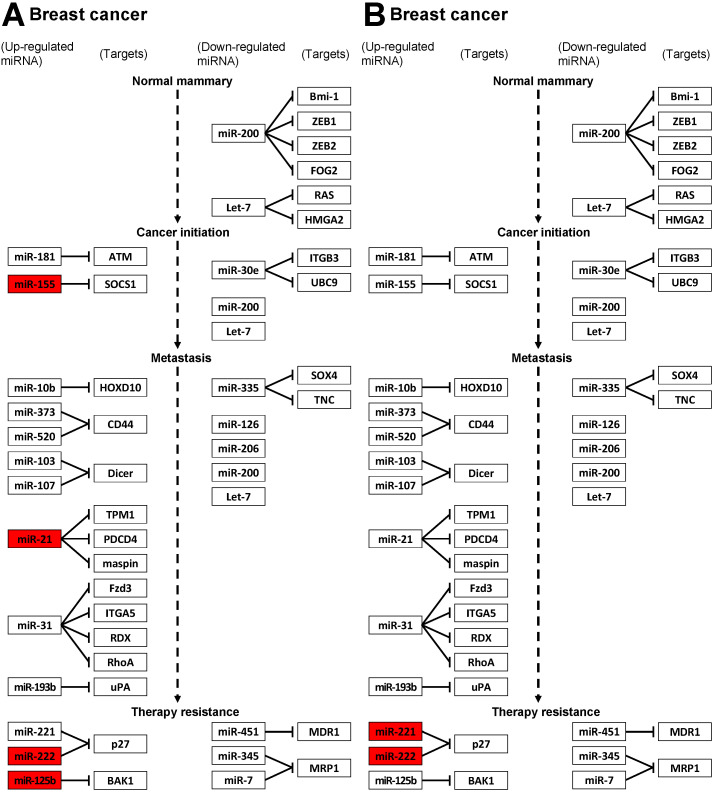
Pathway analysis in breast cancer. Red boxes indicate miRNAs detected in the pathway among DE-miRNAs. (**A**) BCMT-T vs. BCMT-N; (**B**) MCMT-T vs. MCMT-N.

We performed the pathway analysis from KEGG based on DE-miRNAs. Among the various pathways in KEGG, we identified the role of miRNAs as “MicroRNAs in cancer”. This category contains sub-pathways for nine different types of cancer. Among these nine types, we focused on the breast cancer pathway. Since only one miRNA was detected in the MCMT-T vs. BCMT-T, this comparison was excluded from the pathway analysis. Figure 2 shows the result of breast cancer pathway in “MicroRNAs in cancer” for the miRNAs of BCMT-T vs. BCMT-N and MCMT-T vs. MCMT-N.

Among the 18 array-based DE-miRNAs of BCMT-T vs. BCMT-N, breast cancer related miRNAs were identified as cfa-miR-21, cfa-miR-129b, cfa-miR-155, and cfa-miR-222. Among the 20 miRNA of MCMT-T vs. MCMT-N, breast cancer related miRNAs were identified as cfa-miR-221 and cfa-miR-222. Moreover, we discovered the expression pattern of target genes of breast cancer related miRNAs identified in the pathway at the mRNA level. The target genes in the BCMT group were BAK1, CDKN1B, PDCD4, SERPINB5, SOCS1, and TPM1, and the target gene in the MCMT group was CDKN1B. Table 1 shows the fold change and *p*-value of target genes at the mRNA level. It was found that the target genes of miRNA were downregulated in the breast cancer pathway; the genes with significant *p*-value under 0.05 at the mRNA level were downregulated (PDCD4, SOCS1, and CDKN1B).

**Figure 3:**
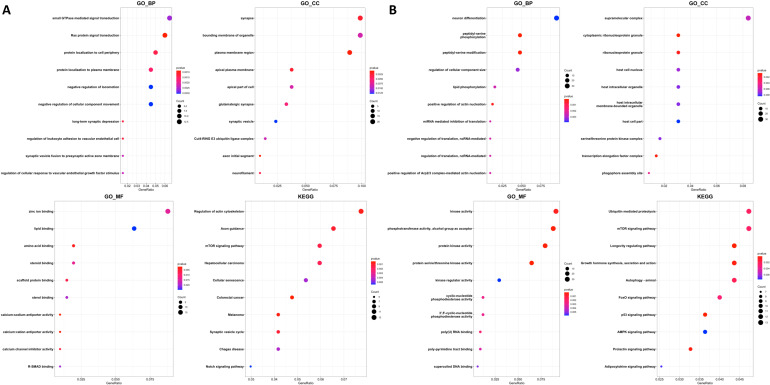
Enrichment analysis for GO_BP, GO_CC, GO_MF, and KEGG. GO_BP: Biological processing; GO_CC: Cellular component; GO_MF: Molecular function of Gene Ontology; KEGG: Biological pathway. (**A**) BCMT-T vs. BCMT-N; (**B**) MCMT-T vs. MCMT-N.

We performed gene-level analysis of DE-miRNAs based on the miRNA-gene associations collected from microRNA target prediction database (miRDB). The miRDB contains information regarding predicted target genes and predicted score for miRNAs. We filtered associations with a predictive score of >90 for miRNA-gene relationships. Since the same gene is associated with multiple miRNAs, the collected target genes were used after de-duplication. Three hundred and seventy target genes in DE-miRNA of BCMT-T vs. BCMT-N, six hundred and forty eight target genes in DE-miRNA of MCMT-T vs. MCMT-N, and three target genes in DE-miRNA of BCMT-T vs. MCMT-T were collected. Functional analysis was performed using clusterProfiler based on predicted genes. Among the various biological functions, we focused on specific biological functions: Autophagy, response to oxidative stress, and p53 signaling pathway. As few target genes were detected in the MCMT-T vs. BCMT-T, this comparison was excluded from the enrichment analysis. Figure 3 shows the results for the top 10 categories among Gene Ontology and KEGG enrichment analysis.

**Figure 4:**
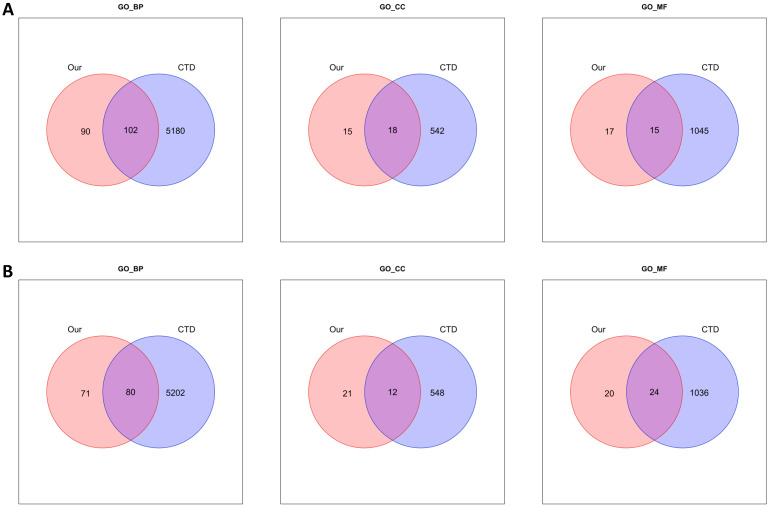
Venn diagrams showing GO_BP, GO_CC, and GO_MF of miRNAs. GO_BP: Biological processing; GO_CC: Cellular component; GO_MF: Molecular function of Gene Ontology; (**A**) BCMT-T vs. BCMT-N; (**B**) MCMT-T vs. MCMT-N.

To evaluate the results of clusterProfiler, we collected disease related Gene Ontologies from the Comparative Toxicogenomics Database (CTD) [24]. We used phenotype (GO)-disease inference networks among various information in CTD, which contains a list of diseases associated with Gene Ontology. We collected a list of GO categories associated with breast cancer using the MESH ID (D001943). In addition, GOs that do not have information on genes from the collected list were removed. We filtered 5282 categories in GO_BP, 560 in GO_CC, and 1060 in GO_MF. We compared GOs with *p*-value < 0.05 among clusterProfiler and filtered CTD (Figure 4).

It was found that the common function of the array-based DE-miRNAs’ target genes was similar to those associated with breast cancer. In particular, categories related to angiogenesis were detected in the intersection. According to Nishida et al., the onset of cancer is related to angiogenesis [25]. Moreover, it was identified that angiogenesis is closely related to progression in breast cancer [25,26]. Significant pathways related breast cancer, such as melanoma (*p*-value < 0.001), breast cancer (*p*-value = 0.009), and microRNAs in cancer (*p*-value = 0.009) in the BCMT group, p53 signaling pathway (*p*-value < 0.001), autophagy, and EGFR tyrosine kinase inhibitor resistance in MCMT group, were observed from KEGG analysis results.

**Figure 5:**
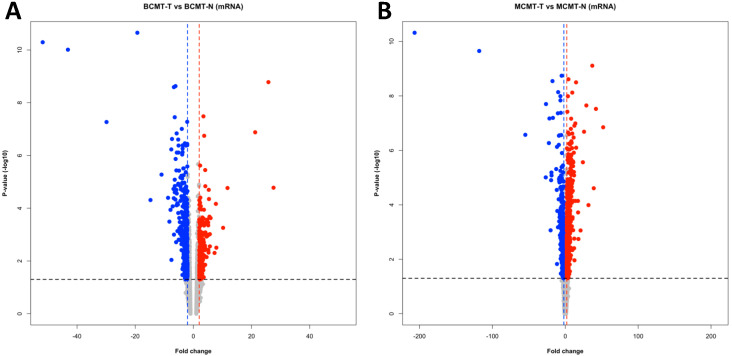
Volcano plot in canine mammary gland tumor for mRNA. Red dots indicate upregulation, blue dots indicate downregulation and grey dots indicate unsignificant miRNAs. Red and blue dotted line are threshold of fold change and black dotted line is threshold of *p*-value. (**A**) BCMT-T vs. BCMT-N; (**B**) MCMT-T vs. MCMT-N.

We extracted mRNA level DEG for a similar comparative analysis as performed using miRNAs. The cut-off values were |fold change| ≥ 2 and *p*-value < 0.05. About 962 genes (392 upregulated and 570 downregulated) in BCMT-T vs. BCMT-N, and 1541 genes (856 upregulated and 685 downregulated) in MCMT-T vs. MCMT-N were identified. Figure 5 shows the volcano plot for identifying DEGs of each comparison based on fold change and *p*-value.

**Figure 6:**
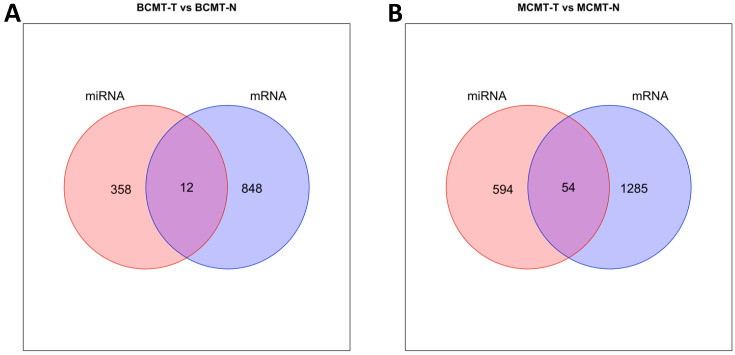
Venn diagram of commonly appearing genes between miRNAs and their targeted mRNAs. (**A**) BCMT-T vs. BCMT-N; (**B**) MCMT-T vs. MCMT-N.

The relationship between identified DEGs and target genes of miRNA was analyzed. Genes corresponding to protein coding were filtered from DEGs. Figure 6 shows the DE-miRNA target gene and DEG of mRNA-based Venn diagram.

The number of genes commonly appearing between miRNA and mRNA was 12 in BCMT group and 54 in MCMT group. It was found that a small number of genes appeared as intersections compared with the number of detected genes in the miRNA and mRNA DEG analysis. To evaluate whether the genes corresponding to the intersection are breast cancer related genes, we collected disease-gene associations from DisGeNet [27]. DisGeNet is a database containing disease-gene associations for humans. Due to the lack of information on disease related genes for dogs, DisGeNet was used to verify genes corresponding to intersections. We identified 10 breast cancer related genes among 12 corresponding to the intersection in BCMT-T vs. BCMT-N (83%), and 25 related genes among 54 corresponding to the intersection in MCMT-T vs. MCMT-N (46%) (Table 2). We detected that the target genes of miRNA showed a significant expression pattern even at the mRNA level. All of the analyzed data are addressed in Supplementary Data S2.

## 3. Discussion

When comparing BCMT-N and BCMT-T, 18 miRNAs were classified as DE-miRNAs with |fold change| ≥ 2 and *p*-value < 0.05. Moreover, cfa-miR-21 and cfa-miR-502 were already discovered for their overexpression in CMT and are diagnostic targets of CMT [23,28,29]. In particular, cfa-miR-21 is related to the inhibition of tumor cell apoptosis in dogs [23]. Human homolog genes of the five DE-miRNAs viz. hsa-miR-21, hsa-miR-185, hsa-miR-125b, hsa-miR-500, and hsa-miR-502 are all related to human cancers, whereas all of the miRNAs except for hsa-miR-500 are related to human BC [30,31,32].

Hsa-miR-21 is a well-known oncomir [19,33] that shows an increase in copy number in human tumor tissues [34]; it is indicated in tumorigenesis, apoptosis, cell proliferation, and cancer progression in human cancers [33]. Since hsa-miR-21 is the only miRNA overexpressed in six types of human cancers, it qualifies as an important candidate for cancer studies [30]. Furthermore, hsa-miR-21 contributes to the maintenance of malignant phenotypes in certain cancers; therefore, it could be used as a biomarker for malignancy [35]. Most importantly, the role of hsa-miR-21 in BC has also been studied; it is correlated with the presence and progression of BC, as it targets the tumor suppressor protein, Programmed Cell Death 4 [36,37]. The overexpression of cfa-miR-21 is observed in both canine (CMT) and human tissue (BC) [23]. In dogs, cfa-miR-21 is attributed to the inhibition of tumor cell apoptosis [23]; it is quite natural that one of the top five DE-miRNAs was found to be cfa-miR-21 in this study (Supplementary Data S1A). However, in contrast to hsa-miR-21 which is identified as a biomarker for tumor malignancy in humans, cfa-miR-21 was found only in the DE-miRNA list of BCMT and not from the MCMT tissue (Data S1A,B).

The function of hsa-miR-185 as a tumor suppressor has been established by various studies; it inhibits the proliferation of human colon cancer cell as well as the development of glioma by inhibiting global DNA methylation [38,39]. The role of hsa-miR-185 in human BC has also been identified as a tumor suppressor since it inhibits BC by regulating S100A8/A9, NF-κB/Snail signaling pathway, and programmed cell death. Although cfa-miR-185 was found to be related to IL-7R expression in dogs [40], its association with tumors has not yet been established. As observed in this study, BMGT individuals showed significantly higher expression of cfa-miR-185 in tumor tissue than normal tissues, which is contrary to the role of tumor suppression shown by hsa-miR-185 in humans.

Moreover, cfa-miR-125b is attributed to host cell resistance against canine influenza virus in [41] and to testicular retinoic acid induced spermatogenesis [42]. However, the role of cfa-miR-125b in canine tumors has not yet been identified; this study is the first to record the overexpression of cfa-miR-125b in MGMT dogs. On the contrary, hsa-miR-125b has been known to act as both an oncogene and a tumor-suppressor gene. Hsa-miR-125b contributes to the regulation of glycolysis, apoptosis, metastasis, and cancer stem cells [43]. Decreased hsa-miR-125b contributes to prostate tumorigenesis via tumor cell behavior alteration as it works as a tumor suppressor [44]. Furthermore, hsa-miR-125b plays a role in γ-irradiation sensitivity in BC; its increased expression results in enhanced apoptotic activity and senescence after irradiation of BC cells [45].

In this study, tumor tissue of MCMT patients showed a significant upregulation of cfa-miR-500 compared with the normal tissue (Data S1B). Since there are no existing reports on the expression patterns or the function of cfa-miR-500 in dogs, the results obtained in this study are of significance to CMT research. In humans, the expression pattern and function of hsa-miR-500 have been studied extensively. Jiang et al. revealed that hsa-miR-500 suppresses the proliferation and metastasis of non-small cell lung cancer. However, most of the studies show the function of the hsa-miR-500 as an oncomir, such as the cfa-miR-500 in this study [46]. Furthermore, hsa-miR-500 has been highly correlated with the malignant progression of gastric cancer [47] and is found to be upregulated in human hepatocellular carcinoma and prostate cancer tissues [48,49].

Hsa-miR-502 is known for its suppressive action on the proliferation of BC [50]. Moreover, it plays a role in inhibiting proliferation, tumor growth, invasion, and metastasis in hepatocellular carcinoma [51]. On the other hand, hsa-miR-502 is observed to function as an oncomir as it promotes cancer cell proliferation and inhibits apoptosis in esophageal cancer [52]. According to Xiaoli et al., cfa-miR-502 is significantly upregulated in CMT compared with normal tissue [29]. Since overexpression of cfa-miR-502 was detected in this study, the role of cfa-miR-502 could be further explored in future studies (Data S1A).

In addition, hsa-miR-146a is associated with diverse tumors in humans, and it works as tumor suppressor miRNA or oncomiR depending on the target gene [53]. Moreover, hsa-miR-146a showed significantly lower expression than normal tissue in gastric cancer tissue, which makes the miRNA an independent prognostic factor for cancer patients [54]. As a tumor suppressor gene, it plays an important role in the proliferation and oncogenic transformation of myeloid cells; it is also found to be downregulated in hepatocellular carcinoma tissue [55,56]. Furthermore, cfa-miR-146a in dogs is expressed in response to a tumor (Data S1A,B) and inflammation similar to hsa-miR-146a in humans; it is one of the overexpressed miRNAs in canine meningioma and is related to cell proliferation and migration [57]. Significantly increased expression of cfa-miR-146a in tumor tissues of MCMT was observed in this study (Data S1B); cfa-miR-146a also contributes to the inflammatory response in canine meningioma and peri-implantitis, as similarly found in humans [57,58].

Nevertheless, cfa-miR-23b has not been mentioned in officially published studies, which makes its increased expression in tumor tissues of MCMT in this study the first to be reported in dogs (Data S1B). However, hsa-miR-23b has been discussed in various studies in relation to human disease; hsa-miR-23b is an oncomir in human BC [59], while it exhibits decreased expression in pituitary adenoma as hsa-miR-23b inhibits proliferation by cell cycle arrest [60]. In addition, hsa-miR-23b is more often identified as a tumor suppressor in human biology and hsa-miR-23b is related to cell aggressiveness inhibition, which indicates its potential use as a biomarker for diagnosis and prognosis of cancer [61]. Hsa-miR-23b inhibits cell proliferation and invasion in prostate cancer, thereby affecting the epithelial-mesenchymal transition process [62]. The downregulation of hsa-miR-23b is related to the poor prognosis of colorectal cancer [63] and cervical cancer [64].

Hsa-miR-221 and hsa-miR-222 miRNAs affect proliferation, differentiation, and invasion of cancer cells, and are upregulated in human BC, multiple myeloma, malignant melanoma, glioma, colorectal cancer, etc. [65,66,67]. The abnormal expression of hsa-miR-221 and hsa-miR-222 is attributed to the development of malignant tumors [67]. Their functions have been studied in detail in human BC. Hsa-miR-221 and hsa-miR-222 affect cancer development and progression as they are related to the telomerase activity, apoptosis, angiogenesis, proliferation, autophagy, and epithelial-mesenchymal transition; they also affect anticancer drug resistance in BC [65,68]. However, in contrast to humans, only a limited number of studies were conducted with cfa-miR-221 and cfa-miR-222. These miRNAs are upregulated in canine prostate cancer, contribute to cell proliferation, and exhibit increased expression in the pituitary when exposed to chronic stress stimulation [62,69]. To date, no study has been conducted in relation to CMT, and the significant upregulation of cfa-miR-221 and cfa-miR-222 in MCMT observed in this study is the first one to be recorded (Data S1B).

A comparison of malignant and benign tumor tissues (MCMT-T vs. BCMT-T) identified cfa-miR-503 as the only DE-miRNA (Figure 1C and Data S1C). In the previous studies, cfa-miR-503 has been found to contribute to follicular growth and oocyte maturation in dogs [70], and to the doxorubicin sensitivity in tumor tissue [71]. On the other hand, in humans, hsa-miR-503 is indicated as a tumor suppressor and oncogenic miRNA. Hsa-miR-503 inhibits tumorigenesis, progression, proliferation, metastasis in hepatocellular carcinoma, lung cancer, BC, colorectal cancer, ovarian cancer, cervical cancer, etc., and functions as a tumor suppressor [72,73,74]. However, these characteristics are not yet studied in dogs.

In summary, among the miRNAs discussed here, we first reported that cfa-miR-503 was related to the malignancy. Although not evaluated in this study, cfa-miR-503 could not only be a novel short RNA-based drug for canine malignant CMTs that functions as a tumor suppressor with anti-metastasis activity, but could also be a novel biomarker for malignancy diagnostic methods in CMT, while the histopathological assessments are vague. Nevertheless, further studies on the mechanism of action should be conducted for the evaluation and validation of cfa-miR-503; this miRNA may be a promising candidate both for a novel drug and a biomarker.

## 4. Materials and Methods

### 4.1. Tissue Sample Collection

Tissue samples were collected from dogs at Seoul National University Veterinary Medical Teaching Hospital as well as from over 10 other local animal hospitals in the Republic of Korea during 2020–2021. CMT diagnosis and malignancy classification were carried out based on both histopathological examination (IDEXX, Seongnam-si, Republic of Korea) and metastasis status. Eight normal tissues and twelve benign CMT (BCMT) tissues were collected from twelve dogs with BCMT (Table 3). Similarly, four normal tissues and eight malignant CMT (MCMT) tissues were collected from eight dogs with MCMT (Table 3). Blood tests were performed for all animals to rule out other medical issues. Normal, unaffected tissues were also collected adjacent to the CMT mass. All samples for miRNA-seq and RNA-Seq were preserved in RNAlater (Thermofisher, Waltham, MA, USA) and stored frozen at −80 °C until further analysis. Samples were collected only after receiving consent from the dog owners. This study was approved by the Seoul National University Institutional Animal Care and Use Committee (approval number: SNU-200217-3-2).

### 4.2. Total RNA Extraction and Quality Check

Total RNA was extracted from each tissue sample using Easy-Spin Total RNA Extraction kit (Intron Biotechnology, Seoul, Republic of Korea) according to the manufacturer’s protocol. RNA purity and integrity were evaluated using ND-2000 Spectrophotometer (NanoDrop, Wilmington, DE, USA) and Agilent 2100 Bioanalyzer (Agilent Technologies, Palo Alto, CA, USA), respectively.

### 4.3. Microarray Hybridization and Scanning for miRNA

Microarray hybridization was performed using the Affymetrix GeneChip miRNA 4.0 Array (Thermofisher, MA, USA) according to the manufacturer’s protocol. Total RNA samples were labeled using FlashTag™ Biotin RNA Labeling Kit (Genisphere, PA, USA). The labeled RNA samples were then quantified, fractionated, and hybridized to the microarray according to the manufacturer’s protocol. RNA-array hybridization was performed on an Affymetrix^®^ 450 Fluidics Station (Thermofisher, MA, USA). The arrays were stained using a GeneChip Fluidics Station 450 (Affymetrix, Santa Clara, CA, USA) and scanned using an Affymetrix GCS 3000 scanner (Affymetrix, Santa Clara, CA, USA). The miRNA-mRNA hybridization signals were analyzed using the Affymetrix^®^ GeneChip™ Command Console.

### 4.4. Raw Data Preparation and Statistical Analysis

Raw data were extracted automatically through the Affymetrix data extraction protocol using the Affymetrix GeneChip^®^ Command Console^®^ Software (AGCC) version 6.1 (ThermoFisher, MA, USA). The CEL files import, miRNA level RMA + DABG-All analyses and results were exported using Affymetrix^®^ Power Tools (APT) Software version 2.11.4 (ThermoFisher, MA, USA). Array data were filtered using probes of annotated species.

### 4.5. RNA-Seq Library Construction and Sequencing

Total RNA concentration was calculated using Quant-IT RiboGreen (Invitrogen, ThermoFisher, MA, USA, #R11490). To assess the integrity of the total RNA, samples were carried out on the TapeStation RNA ScreenTape (Agilent Technologies, CA, USA, #5067-5576). Only high-quality RNA preparations with RNA integrity number (RIN) > 7.0 were used for RNA library construction. A library was independently prepared with 1 µg total RNA of each sample using the Illumina TruSeq Stranded mRNA Sample Prep Kit (RS-122-2101, Illumina, Inc., San Diego, CA, USA). The first step in the workflow involved purifying the poly-A containing mRNA molecules using poly-T-attached magnetic beads. Following purification, the mRNA samples were fragmented into small pieces using divalent cations under an elevated temperature. The cleaved RNA fragments were then used as templates to generate first strand cDNA using SuperScript II reverse transcriptase (Invitrogen, MA, USA)) with random primers. This was followed by second strand cDNA synthesis using DNA Polymerase I, RNase H, and dUTP. Next, the generated double stranded cDNA fragments were subjected to an end repair process involving adenylation followed by adapter ligation. Thereafter, the products were purified and enriched with PCR to create the final cDNA library. The libraries were quantified using KAPA Library Quantification kits for Illumina Sequencing platforms according to the qPCR Quantification Protocol Guide (KAPA BIOSYSTEMS, Wilmington, DE, USA) and qualified using the TapeStation D1000 ScreenTape (Agilent Technologies, CA, USA). Finally, indexed libraries were submitted to Illumina NovaSeq (Illumina, Inc., San Diego, CA, USA) to perform paired-end (2 × 100 bp) sequencing.

### 4.6. Data Analysis

At the miRNA level, raw data were extracted automatically in Affymetrix data extraction protocol using the software provided by Affymetrix GeneChip^®^ Command Console^®^ Software (AGCC). The CEL files import, miRNA level RMA + DABG-All analyses and results were exported using Affymetrix^®^ Power Tools (APT) Software. Array data were filtered using probes of annotated species.

At the mRNA level, we preprocessed the raw reads from the sequencer to remove low quality and adapter sequences before analysis and aligned the processed reads to the Canis lupus familiaris (CanFam3.1) database using HISAT v2.1.0 [75]. HISAT utilizes two types of indexes for alignment (a global, whole-genome index and tens of thousands of small local indexes). These two types of indexes are constructed using the same Burrows–Wheeler transform (BWT), which is a graph FM index (GFM), such as Bowtie2. Due to its use in efficient data structures and algorithms, HISAT generates spliced alignments several times faster than Bowtie and BWA. The reference genome sequence of Canis lupus familiaris (CanFam3.1) and annotation data were downloaded from the NCBI database. Then, the transcript assembly of known transcripts was processed by StringTie v2.1.3b [76]. Based on the results, expression abundance of transcripts was calculated as read count or Fragments Per Kilobase of exon per Million fragments mapped (FPKM value) per sample. The expression profiles were used for additional analysis, such as differentially expressed genes (DEGs). In groups with different conditions, DEGs or transcripts can be filtered through statistical hypothesis testing.

A comparative analysis between test and control samples was carried out using independent t-test and fold change with the null hypothesis, which indicates that no difference exists among groups. False discovery rate (FDR) was controlled by adjusting *p*-value using Benjamini-Hochberg algorithm. All statistical tests and visualization of DEGs were conducted using R statistical language (v3.3.2).

KEGG Mapper [77] and clusterProfiler v3.18.1 [78] in R were used to perform the functional analysis of miRNAs and genes. Based on KEGG Mapper, the roles of miRNAs and genes were discovered. Biological functions of DEGs were analyzed based on clusterProfiler. We collected miRNA-gene associations from miRDB [79] and performed gene level analysis based on miRNA targets. Moreover, DEGs were identified by comparisons at the mRNA level; additional analysis was performed between the miRNA target genes and DEGs at mRNA levels.

## 5. Conclusions

In this study, we identified multiple BC-related DEGs in CMT samples. Although several miRNAs were significantly altered by tumorigenesis, only cfa-miR-503 was differentially expressed by malignancy. Along with our results, we conclude that cfa-miR-503 could be used as a potential biomarker for diagnosis and prognostic evaluation of malignant CMTs. Moreover, it could be suggested as a novel RNA-based drug to alleviate metastasis and proliferation of malignant CMTs.

## Figures and Tables

**Table 1:** The mRNA expression profile of miRNA-target genes.

BCMT-T vs. BCMT-N		
Gene	Fold Change	*p*-Value
BAK1	1.134	0.390
CDKN1B	−1.256	0.167
PDCD4	−2.486	<0.001
SERPINB5	1.081	0.838
SOCS1	−2.183	0.005
TPM1	−1.249	0.177
**MCMT-T vs. MCMT-N**		
**Gene**	**Fold Change**	***p*-Value**
CDKN1B	−1.612	0.025

**Table 2:** Intersection and breast cancer related genes of miRNA and mRNA. Bold type indicates breast cancer related gene.

Comparison	Genes
BCMT-T vs. BCMT-N	ACSM5, **ALDH1A1**, **ANTXR2**, **F2RL2**, **FOS**, **KLF4**, **LEF1**, **NOVA1**, **PDGFRB**, **RORA**, SDK1, **TRIM46**
MCMT-T vs. MCMT-N	**ALDH1A2**, **ANK2**, **APLN**, B3GNT7, BEND6, **CHEK1**, CHST1, **CKAP4**, DCBLD2, **DCLK1**, ECSCR, **ELF5**, ELL2, FAM81A, **FBXO5**, **FHL1**, **GJA1**, GJC1, GPAM, HSPA4L, **HUNK**, **IGF1**, **JPH1**, KCNQ5, KLHDC1, **LONRF3**, **MAP3K1**, MEGF10, **MMD**, **MMP13**, MYBL1, NCKAP5, **NOVA1**, NUAK2, PCOLCE2, **PGAM1**, PRUNE2, **PTPRD**, RIMS3, **RORA**, RTKN2, SEC23A, SEMA6D, **SEMA7A**, SESN1, SIMC1, **SLC6A6**, **SLC7A11**, ST6GALNAC3, TEF, TMEM51, TMOD2, VGLL3, **WASF3**

**Table 3:** Animals used for sample collection.

Tumor Classification	ID	Breed	Sex	Age (Years)	Histopathological Features	Normal Tissue
Benign CMT	BCMT-1	Shih-tzu	F *	7	Benign mixed mammary tumor	Y
	BCMT-2	Alaskan malamute	F	6	Benign mammary complex adenoma	Y
	BCMT-3	Shih-tzu	FS **	11	Benign mixed mammary tumor	Y
	BCMT-4	Toy poodle	FS	12	Mammary adenoma	Y
	BCMT-5	Maltese	F	10	Simple adenoma	Y
	BCMT-6	Bichon fries	F	6	Benign mammary lobular hyperplasia	Y
	BCMT-7	Dachshund	F	11	Complex mammary adenoma	Y
	BCMT-8	Mixed	FS	16	Benign mammary adenoma	Y
	BCMT-9	Maltese	FS	11	Complex mammary adenoma	N
	BCMT-10	Cocker spaniel	F	10	Complex mammary tubular adenoma	N
	BCMT-11	Maltese	FS	14	Benign mammary complex adenoma	N
	BCMT-12	Poodle	FS	11	Benign mammary complex adenoma	N
Malignant CMT	MCMT-1	Pomeranian	F	12	Mammary ductular adenocarcinoma, low grade (grade 1)	Y
	MCMT-2	Maltese	F	12	Mammary adenocarcinoma, high grade	Y
	MCMT-3	Shih-tzu	FS	11	Tubulopapillary mammary gland carcinoma, low grade (grade 1)	Y
	MCMT-4	Beagle	FS	11	Mammary ductular adenocarcinoma, low grade (grade 1)	N
	MCMT-5	Mixed	F	8	Multinodular mammary gland adenocarcinoma	Y
	MCMT-6	Poodle	FS	11	Mammary ductular adenocarcinoma, low grade (grade 1)	N
	MCMT-7	Poodle	FS	13	Mammary adenocarcinoma, high grade	N
	MCMT-8	Shih-tzu	F	11	Mammary carcinoma, complex type (grade 1)	N

* F: Female; ** FS: Spayed Female; Y: Collected; N: Not Collected.

## Data Availability

Not applicable.

## Supplementary Materials

The following supporting information can be downloaded at: https://www.mdpi.com/article/10.3390/ijms24032618/s1.

## Abbreviations

CMT: Canine mammary gland tumor; BC: Breast cancer; p53: Tumor protein 53; BRCA1: Breast cancer1; BRCA2: Breast cancer2; PTEN: Phosphatase and tensin homolog; STK11: Serine/threonine kinase11; DE-miRNAs: Differentially expressed miRNAs; BCMT: Benign CMT; MCMT: Malignant CMT; RIN: RNA integrity number; DEGs: Differentially expressed genes; miRDB: MicroRNA target prediction database; CTD: Comparative toxicogenomics database.
